# An Efficient and Facile Methodology for Bromination of Pyrimidine and Purine Nucleosides with Sodium Monobromoisocyanurate (SMBI)

**DOI:** 10.3390/molecules181012740

**Published:** 2013-10-15

**Authors:** Jyotirmoy Maity, Roger Stromberg

**Affiliations:** Department of Biosciences and Nutrition, Karolinska Institute (KI), Novum, Huddinge SE-141 83, Sweden

**Keywords:** bromination, sodium monobromoisocyanurate (SMBI), pyridine-based nucleosides, purine-based nucleosides, bromonucleosides

## Abstract

An efficient and facile strategy has been developed for bromination of nucleosides using sodium monobromoisocyanurate (SMBI). Our methodology demonstrates bromination at the C-5 position of pyrimidine nucleosides and the C-8 position of purine nucleosides. Unprotected and also several protected nucleosides were brominated in moderate to high yields following this procedure.

## 1. Introduction

Over the past decades, 5-halopyrimidine nucleosides have been found to be of interest for their antiviral [1,2,3] and antineoplastic [4,5] properties. 5-Bromo-2'-deoxyuridine has shown *in vivo* antiviral activity through its incorporation into the DNA of replicating cells as a structural analogue of thymidine [6]. Radiolabeled 5-bromo- and 5-iodo-pyrimidine nucleoside analogues have been tested for their potential in diagnostic oncology [7]. In search of new potential agents with superior biological activity, a variety of structural and functional moieties have been introduced into pyrimidine and purine nucleoside analogues by palladium assisted routes [8]. Halonucleosides have been used extensively as starting material for these reactions. 8-Bromopurine nucleosides have been used as key intermediates for synthesizing fluorescently labeled nucleosides, which have promising applications in the field of molecular biology [9,10]. In view of this relevance, development of efficient methodology for the synthesis of 5-bromopyrimidine and 8-bromopurine nucleosides is always of immense interest.

Direct halogenation of nucleosides has been found to be a proficient approach for the synthesis of 5-halopyrimidine, 6-halopurine [11,12,13] and 8-halopurine nucleosides [14]. Unprotected and protected nucleosides have been halogenated by direct reaction with halogens or halogenating agents. Bromination at C-5 of pyrimidine moieties was achieved by reaction of Br_2_/water [15], Br_2_/DMF [16], Br_2_/CCl_4_ [17], Br_2_/CCl_4_ in solvent mixture of anhydrous acetic acid and pyridine [18], *N*-bromosuccinimide (NBS) in DMF [19], NBS/NaN_3_ in DME [20], NBS in ionic liquids [21], *m*-chloroperbenzoic acid (MCPBA)/HBr in DMA or DMF [22], ceric ammonium nitrate (CAN)/LiBr in AcOH or MeCN [23], KBr/potassium monoperoxysulfate under aqueous conditions [24]. Bromination of C-8 of purine moieties has been attained by reaction of Br_2_ in glacial AcOH/NaOAc [25], NBS in DMF [19], Br_2_ in NaOAc buffer/dioxane [10] and Br_2_ in NaOAc buffer [26,27]. A thorough study of the literature procedures for bromination of nucleosides shows that most of these methods have some disadvantages, such as highly acidic reaction condition, handling of toxic reagents, longer reaction time, complicated work up procedure and low yield of the products. Noticeably, very few of these methods are general for bromination of pyrimidine nucleosides as well as purine nucleosides.

In recent years, sodium monobromoisocyanurate (SMBI) has proven its capacity as an efficient brominating agent. It has been used in various solvents to brominate a range of aromatic moieties with both activating and deactivating substituents [28]. Bromination of azulene derivatives with SMBI was achieved in DCM and DCM/water [29]. Unprotected aromatic amino acids were also brominated with SMBI in aq. H_2_SO_4_ to produce mono-brominated products in good yield [30]. SMBI has also been found to act as a catalyst for the synthesis of aryl thiocyanates [31]. Following the literature on SMBI, we believed it to be an efficient brominating agent for nucleosides. In this report, we describe the ability of SMBI to brominate the C-5 of pyrimidine nucleosides and C-8 of purine nucleosides. Along with unprotected nucleosides of naturally occurring bases, we have also successfully brominated nucleosides with alkyl, silyl, dimethoxytrityl (DMTr)/monomethoxytrityl (MMTr) protection on the sugar moiety and/or acyl protection on the nucleobase. DMTr/ MMTr groups are among the most frequently used protective groups in nucleoside chemistry. Due to their high sensitivity towards acidic reaction conditions, there is no literature procedure available for bromination of DMTr/MMTr protected nucleosides. Here we report for the first time in literature bromination of DMTr/MMTr protected nucleosides.

## 2. Results and Discussion

**Scheme 1:**
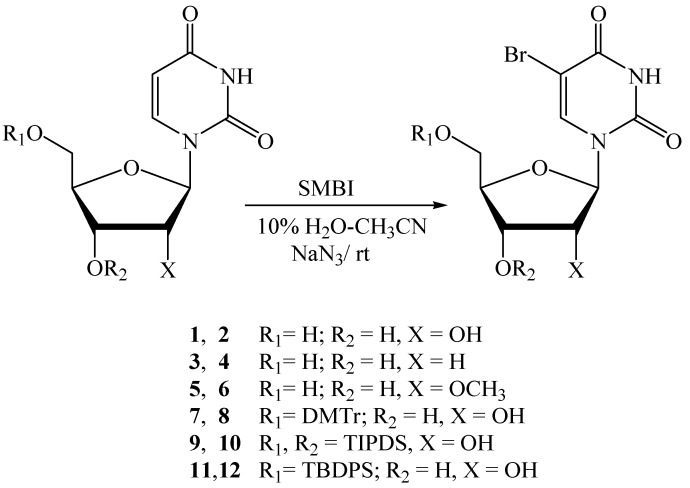
Bromination of uridine and derivatives thereof.

Initially, we tried to brominate the C-5 position of uridine (**1**) and screened various reaction conditions (Scheme 1, Table 1). Treatment of **1** with SMBI (1.05 equiv.) in a solvent mixture of 10% H_2_O-CH_3_CN in presence of NaN_3_ (4.0 equiv.) at room temperature produced 5-bromouridine (**2**) [20,21,23,32] in 94% yield within 30 minutes. Next, we tried another reaction with **1**, where we added 2.0 equiv. of NaN_3_ keeping other conditions unaltered. Bromo nucleoside **2** was obtained in 88% yield in 2 h by this reaction. However, when **1** was treated with SMBI (1.15 equiv.) in 10% H_2_O-CH_3_CN in the absence of NaN_3_ at room temperature, the bromination reaction was slow and took a longer time (18 h) to afford **2** in only 40% yield.

**Table 1:** Screening for appropriate bromination condition of uridine and bromination of uridine derivatives.

Entry	Substrate	Product	SMBI (eq.)	NaN_3_ (eq.)	Solvent	Reaction time (h)	Yield (%) (NMR/TLC)	Isolated yield (%)
1	**1**	**2**	1.05	4.0	10% H_2_O-CH_3_CN	0.5	94 *^a^*	79 *^b^*
2	**1**	**2**	1.05	2.0	10% H_2_O-CH_3_CN	1.5	88 *^a^*	-
3	**1**	**2**	1.15	0	10% H_2_O-CH_3_CN	18.0	40 *^c^*	-
4	**1**	**2**	1.15	0	20% H_2_O-DMF	18.0	15 *^c^*	-
5	**3**	**4**	1.05	4.0	10% H_2_O-CH_3_CN	1.2	90 *^a^*	86 *^d^*
6	**5**	**6**	1.15	4.0	10% H_2_O-CH_3_CN	1.5	-	93 *^d^*
7	**7**	**8**	1.1	4.0	10% H_2_O-CH_3_CN	1.0	86 *^a^*	81 *^d^*
8	**9**	**10**	1.1	4.0	10% H_2_O-CH_3_CN	1.5	94 *^a^*	91 *^d^*
9	**11**	**12**	1.1	4.0	10% H_2_O-CH_3_CN	1.2	-	92 *^d^*

*^a^* Yield calculated by ^1^H-NMR spectroscopy of the crude reaction mixture. *^b^* Isolated yield after crystallization. *^c^* Yield judged by TLC. *^d^* Isolated yield after purification by column chromatography.

We also investigated bromination of **1** in 20% H_2_O-DMF without addition of NaN_3_. Even after a prolonged reaction time (18 h) bromo-nucleoside **2** was only obtained in low yield. Analysis of these reaction conditions applied for uridine (entry 1/entry 2/entry 3/entry 4, Table 1) verifies the crucial role of NaN_3_ in the halogenation reactions (bromination reactions in our case) at the C-5 position of the pyrimidine nucleosides. The azido group plays the role of an excellent leaving group from the C-6 position of the 5-halo-6-azido-5,6-dihydro intermediates to restore the 5,6-olefinic bond [20]. The bromination reaction conditions optimized for uridine (**1**) were next successfully employed for other uridine derivatives. Bromination of 2'-deoxyuridine (**3**) was carried out with SMBI (1.05 equiv.) and NaN_3_ (4.0 equiv.) in 10% H_2_O-CH_3_CN which produced 5-bromo-2'-deoxyuridine (**4**) [20,21,23] in 90% yield (Table 1). 2'-*O*-Methyluridine (**5**) was treated under the same reaction conditions, followed by addition of 0.1 equiv. of SMBI after 1 h. The reaction was stopped after 1.5 h and purification of the product by column chromatography afforded 5-bromo-2'-*O*-methyluridine (**6**) in 93% yield. Reaction of 5'-*O*-dimethoxytrityluridine (**7**) with 1.1 equiv. of SMBI in 10% H_2_O-CH_3_CN in the presence of NaN_3_ (4.0 equiv.) produced bromo nucleoside **8** [33] in 86% yield. Similar treatment of silyl protected nucleosides 3',5'-*O*-tetraisopropyldisilyl-uridine (**9)** and 5'-*O*-*tert*-butyldiphenylsilyl-uridine (**11)** with 1.1 equivalent of SMBI afforded the corresponding bromonucleosides **10** [34] and **12** in 94% and 92% yields, respectively.

**Scheme 2:**
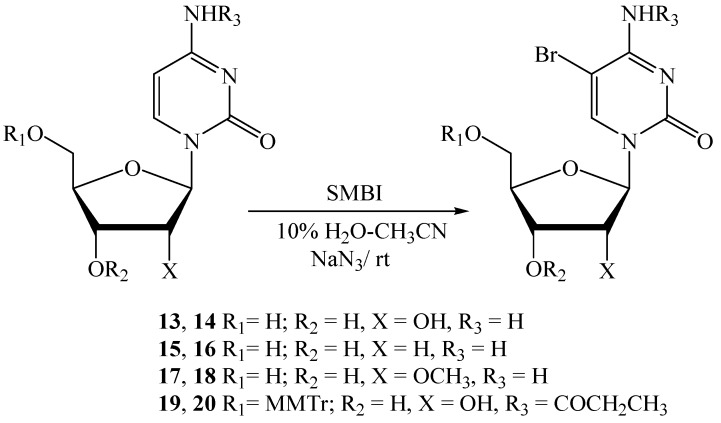
Bromination of cytidine and derivatives thereof.

We then applied our methodology for the bromination of cytidine (**13**) (Scheme 2). Nucleoside **13** was treated with 1.05 equiv. of SMBI in presence of NaN_3_ (4.0 equiv.) in 10% H_2_O-CH_3_CN at rt. The progress of the reaction was followed by TLC. Additional amounts of SMBI (0.2 equiv. each time) were added into the reaction mixture after 1 h and 2 h. This reaction produced 5-bromocytidine (**14**) in 83% yield after 3 h (Table 2). In another reaction, **13** was dissolved in 20% H_2_O-DMF and treated with 1.2 equiv. of SMBI, followed by further additions of the reagent (0.3 equiv. each time) after 3 h and 6 h stirring at rt. The reaction was stirred for 20 h, followed by the work up, which produced the brominated nucleoside **14** in 73% yield. The reaction time and yield for bromination of **13** (entry 1/entry 2, Table 2) were compared, which indicated the first conditions to be more efficient.

**Table 2:** Comparison of suitable bromination condition for cytidine and bromination of cytidine derivatives.

Entry	Substrate	Product	SMBI (eq.)	NaN_3_ (eq.)	Solvent	Reaction time (h)	Yield (%) (NMR)	Isolated yield (%)
1	**13**	**14**	1.45	4.0	10% H_2_O-CH_3_CN	3.0	83 *^a^*	69 *^b^*
2	**13**	**14**	1.8	0	20% H_2_O-DMF	20.0	73 *^a^*	-
3	**15**	**16**	1.6	4.0	10% H_2_O-CH_3_CN	3.5	73 *^a^*	66 *^c^*
4	**17**	**18**	1.25	4.0	10% H_2_O-CH_3_CN	2.0	-	78 *^c^*
5	**19**	**20**	1.25	4.0	10% H_2_O-CH_3_CN	2.5	-	59 *^c^*

*^a^* Yield calculated by ^1^H-NMR spectroscopy of the crude reaction mixture. *^b^* Isolated yield after crystallization. *^c^* Isolated yield after purification by column chromatography.

We then performed a reaction of nucleoside **15** with 1.2 equiv. of SMBI in 10% H_2_O-CH_3_CN and in the presence of NaN_3_. Following the progress of the reaction on TLC, an excess amount of SMBI (0.2 equiv. each time) was added after 1.5 h and 3 h. 5-Bromo-dC, **16**, was obtained in 73% yield after 3.5 h stirring at rt. 2'-*O*-Methylcytidine (**17**) was treated with 1.25 equiv. of SMBI (entry 4, Table 3) which produced nucleoside **18** in 78% yield after 2 h. When *N*^4^-propanoyl-5'-*O*-dimethoxytritylcytidine (**19**) [35] was treated with 1.25 equiv. of SMBI, 5-bromo-*N*^4^-propanoyl-5'-*O*-dimethoxytritylcytidine (**20**) was obtained in 59% yield in 2.5 h. (entry 5, Table 2).

**Table 3:** Bromination conditions for purine nucleosides.

Entry	Substrate	Product	SMBI (eq.)	Solvent	Reaction time (h)	Yield (%) (NMR/TLC)	Isolated yield (%)
1	**21**	**22**	1.4	20% H_2_O-DMF	2.5	89 *^a^*	73 *^b^*
2	**23**	**24**	1.3	20% H_2_O-DMF	2.0	93 *^a^*	88 *^d^*
3	**25**	**26**	1.45	20% H_2_O-DMF	2.0	78 *^a^*	71 *^d^*
4	**27**	**28**	1.2	20% H_2_O-DMF	4.0	<5 *^c^*	-
5	**29**	**30**	1.5	20% H_2_O-DMF	4.0	15 *^c^*	-
6	**31**	**32**	1.5	20% H_2_O-DMF	4.0	62 *^a^*	57 *^d^*
7	**33**	**34**	1.25	20% H_2_O-DMF	2.0	-	96 *^d^*

*^a^* Yield calculated by ^1^H-NMR spectroscopy of the crude reaction mixture. *^b^* Isolated yield after crystallization. *^c^* Yield judged by TLC. *^d^* Isolated yield after purification by column chromatography.

**Scheme 3:**
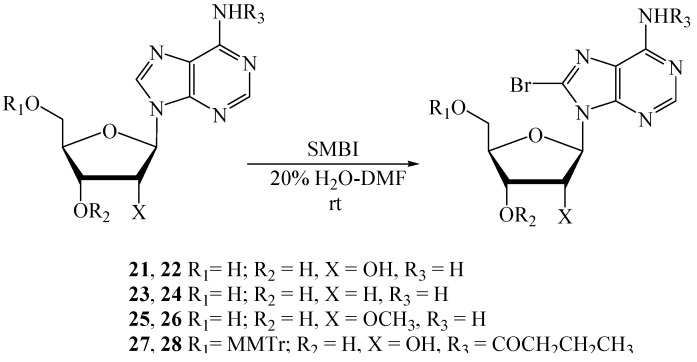
Bromination of adenosine and derivatives thereof.

Unlike the pyrimidine nucleosides, purine nucleosides were not completely soluble in 10% H_2_O-CH_3_CN. This observation led us to carry out the reactions in 20% H_2_O-DMF. Adenosine (**21**) was treated with SMBI (1.1 equiv.) in 20% H_2_O-DMF at rt and the reaction was monitored by TLC (Scheme 3). Additional amounts of the reagent were added (0.2 equiv. after 1 h and 0.1 equiv. after 2 h) to drive the reaction towards completion. Reaction was stopped after 2.5 h to afford the brominated nucleoside **22** [26,32,36] in 89% yield (entry 1, Table 3).

Deoxyadenosine, **23**, was stirred with 1.25 equiv. of SMBI for 1 h followed by addition of 0.5 equiv of the reagent. Reaction was stopped after stirring for another 1 h when compound **24** [10] was obtained in 93% yield. Similarly, 2'-*O*-Methyladenosine (**25**) was treated with SMBI (1.25 equiv.), and the reaction mixture was stirred for 1 h, followed by addition of 0.2 equiv. of the reagent. Reaction was worked up after 2 h, and the bromonucleoside **26** [27] was obtained in 78% yield. However, the 5'-*O*- and base protected nucleoside **27** [35] was found to be unsuitable for bromination under these reaction conditions. We could trace the product by ESI-TOF mass spectroscopy, but it was difficult to isolate from the reaction mixture as most of the starting material remained unreacted.

**Scheme 4:**
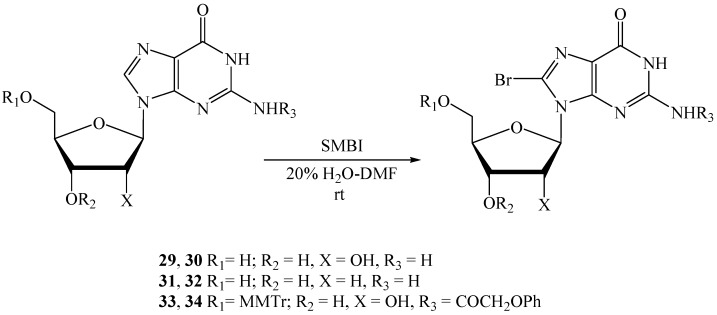
Bromination of guanosine and derivatives thereof.

Next, we extended the application of the SMBI reagent for guanine-based nucleosides. Guanosine (**29**) was treated with SMBI (1.25 equiv.) in 20% H_2_O-DMF at rt, followed by addition of another lot of SMBI (0.25 equiv.) after 2 h (Scheme 4). Reaction was stopped after 4 h to produce 8-bromo-guanosine (**30**) [37], albeit in low yield (entry 5, Table 3).

Similar treatment of deoxyguanosine, **31**, with SMBI (starting the reaction with 1.25 equiv. of reagent and then addition of 0.25 equiv. of the reagent after 2 h stirring) produced 62% of bromo nucleoside **32** [37] in 4 h. We did not get a very high yield for bromination of **29**, so we explored these reaction conditions for a protected guanosine derivative. *N*^2^-Phenyloxyacetyl-5'-*O*-monomethoxytrityl-guanosine (**33**) [35] was treated with 1.1 equiv. of SMBI and stirred at rt. After 1.5 h, a supplementary amount (0.15 equiv.) of the reagent was added to drive forward the reaction. Bromonucleoside **34** was obtained from the reaction in 96% yield after 2 h.

## 3. Experimental

### 3.1. General

All solvents were of commercial analytical grade and reagents were of commercial reagent grade and used without further purification. Anhydrous reactions were carried out under nitrogen atmosphere. ^1^H-NMR (400 MHz) and ^13^C-NMR (100 MHz) spectra were recorded in D_2_O, DMSO-*d*_6_, CD_3_OD or CDCl_3_ on a Bruker DRX 400. Chemical shifts are reported in ppm relative to tetramethylsilane or the deuterated solvent as internal standard for ^1^H and ^13^C-NMR. Coupling constants (*J* values) are given in Hz. Structural assignments are based on DEPT-135 and COSY experiments. HRMS-ESI was recorded on a Micromass LCT in negative ion mode. TLC was run on analytical TLC plates (silica gel 60 F_254_), purchased from Merck (Stockholm, Sweden). Spots were visualized by UV light.

### 3.2. Typical Procedure for the Bromination of Pyrimidine Nucleosides and Their Derivatives

2'-*O*-Methyluridine (**5**, 0.103 g, 0.4 mmol) was dissolved in aqueous acetonitrile solution (H_2_O:CH_3_CN 1:9, 5 mL) under stirring. NaN_3_ (0.104 g, 1.6 mmol) was added, followed by addition of SMBI (0.101 g, 0.44 mmol) at r.t. and the mixture was stirred. Progress of the reaction was followed by TLC. On completion of the reaction after 1.5 h, the reaction mixture was filtered, evaporated to dryness under reduced pressure and coevaporated with acetonitrile (2 × 2 mL). The crude reaction mixture was purified by column chromatography (4%–6% MeOH in DCM, v/v) to afford bromo nucleoside **6** (0.117 g, 93%) in pure form as a white solid.

*5-Bromo-2'-O-methyluridine* (**6**). M.p. Turns brown above 200 °C; *R_f_* = 0.42 (20% methanol in dichloromethane); ^1^H-NMR (DMSO-*d*_6_): δ = 3.39 (s, 3H, OCH_3_), 3.57–3.60 (m, 1H, C-5'H_a_), 3.69–3.73 (m, 1H, C-5'H_b_), 3.81 (t, *J* = 4.4 Hz, 1H, C-2'H), 3.86–3.87 (m, 1H, C-4'H), 4.12 (q, *J* = 5.6 Hz, 1H, C-3'H), 5.12 (d, *J* = 6.4 Hz, 1H, C-3'OH), 5.32 (t, *J* = 4.4 Hz, 1H, C-5'OH), 5.80 (d, *J* = 3.6 Hz, 1H, C-1'H), 8.53 (s, 1H, C-6H), 11.82 (s, 1H, NH). ^13^C-NMR (DMSO-*d*_6_): δ = 57.5 (OCH_3_), 59.5 (C-5'), 67.6 (C-3'), 82.9 (C-2'), 84.7 (C-4'), 86.6 (C-1'), 95.6 (C-5), 140.0 (C-6), 149.6 (C-2), 159.0 (C-4). HRMS-ESI (*m/z*): calcd. for C_10_H_12_BrN_2_O_6_ [M−H]^−^, 334.9884; found 334.9881.

*5-Bromo-5'-O-tert-butyldiphenylsilyluridine* (**12**). White solid (0.103 g, 92%), M.p. Turns black above 185 °C; *R_f_* = 0.35 (5% methanol in dichloromethane); ^1^H-NMR (CD_3_OD): δ = 1.11 (s, 9H, (C(CH_3_)_3_), 3.84 (dd, *J* = 11.6, 3.2 Hz, 1H, C-5'H_a_), 3.98 (dd, *J* = 11.6, 2.4 Hz, 1H, C-5'H_b_), 4.07–4.09 (m, 1H, C-4'H), 4.23–4.28 (m, 2H, C-2'H and C-3'H), 6.02 (d, *J* = 5.6 Hz, 1H, C-1'H), 7.40–7.48 (m, 6H, C-3''H, C-4''H, C-5''H, C-3'''H, C-4'''H and C-5'''H), 7.72 (d, *J* = 7.2 Hz, 4H, C-2''H, C-6''H, C-2'''H and C-6'''H), 7.97 (s, 1H, C-6H). ^13^C-NMR (CD_3_OD): δ = 20.2 (*C*(CH_3_)_3_), 27.7 (C(*C*H_3_)_3_), 65.1 (C-5'), 71.7 (C-2'), 76.1 (C-3'), 86.6 (C-4'), 90.2 (C-1'), 99.3 (C-5), 129.0 (C-3'', C-5'', C-3''' and C-5'''), 131.1 , 131.2 (C-4'' and C-4'''), 133.8 , 134.1 (C-1'' and C-1'''), 136.7 , 136.8 (C-2'', C-6'', C-2''' and C-6'''), 140.0 (C-6), 156.8 (C-2), 167.4 (C-4). HRMS-ESI (*m/z*): calcd. for C_25_H_28_BrN_2_O_6_ [M−H]^−^, 559.0905; found 559.0927.

*5-Bromo-2'-O-methylcytidine* (**18**). White solid (0.052 g, 78%), M.p. 86–89°C; *R_f_* = 0.36 (2.5% methanol in dichloromethane); ^1^H-NMR (D_2_O): δ = 3.49 (s, 3H, OCH_3_), 3.77 (dd, *J* = 9.0, 3.6 Hz, 1H, C-5'H_a_), 3.88–3.91 (m, 1.6H, C-5'H_b(1)_ and C-2'H), 3.94 (d, *J* = 2.4 Hz, 0.4H, C-5'H_b(2)_), 4.01–4.04 (m, 1H, C-4'H), 4.22 (dd, *J* = 7.2, 5.2 Hz, 1H, C-3'H), 5.85 (d, *J* = 2.8 Hz, 1H, C-1'H), 8.27 (s, 1H, C-6H). ^13^C-NMR (D_2_O): δ = 58.7 (OCH_3_), 60.1 (C-5'), 68.0 (C-3'), 83.7 (C-2'), 84.1 (C-4'), 88.9 (C-1'), 89.2 (C-5), 142.5 (C-6), 156.5 (C-2), 163.3 (C-4). HRMS-ESI (*m/z*): calcd. for C_10_H_13_BrN_3_O_5_ [M−H]^−^, 334.0044; found 334.0049.

*5-Bromo-5'-O-monomethoxytrityl-N^4^-propanoyl-2'-deoxycytidine* (**20**). White solid (0.037 g, 59%), M.p. 96–101 °C; *R_f_* = 0.59 (25% methanol in dichloromethane); ^1^H-NMR (CDCl_3_): δ = 1.12 (t, *J* = 7.2 Hz, 3H, COCH_2_C*H*_3_), 2.18–2.27 (m, 1H, C-2'H_a_), 2.43 (br s, 1H, C-3'OH), 2.68–2.71 (m, 1H, C-2'H_b_), 3.03–3.08 (m, 2H, COC*H*_2_CH_3_) , 3.32 (dd, *J* = 10.6, 3.2 Hz, 1H, C-5'H_a_), 3.36 (dd, *J* = 10.6, 3.2 Hz, 1H, C-5'H_b_), 3.73 (s, 3H, OCH_3_), 4.09–4.10 (m, 1H, C-4'H), 4.49 (brs, 1H, C-3'H), 6.19 (t, *J* = 6.4 Hz, 1H, C-1'H), 6.77 (d, *J* = 9.2 Hz, 2H, C-3''H and C-5''H), 7.15–7.24 (m, 8H, C-2''H, C-6''H, C-3'''H, C-4'''H, C-5'''H, C-3''''H, C-4''''H and C-5''''H), 7.33 (d, *J* = 7.6 Hz, 4H, C-2'''H, C-6'''H, C-2''''H and C-6''''H), 7.78 (s, 1H, NHCO), 8.25 (s, 1H, C-6H). ^13^C-NMR (CDCl_3_): δ = 8.5 (COCH_2_*C*H_3_), 32.0 (CO*C*H_2_CH_3_), 42.4 (C-2'), 55.4 (OCH_3_), 63.4 (C-5'), 72.2 (C-3'), 87.0 (C-4'), 87.5 (O*C*(Ar)_3_), 87.8 (C-1'), 88.2 (C-5), 113.5 (C-3'' and C-5''), 127.4 (C-4''' and C-4''''), 128.4 (C-2'', C-6'', C-3''', C-5''', C-3'''' and C-5''''), 130.5 (C-2''', C-6''', C-2'''' and C-6''''), 135.0 (C-1''), 143.5 (C-1'''), 143.9 (C-1''''), 144.0 (C-6), 153.5 (C-2), 157.2 (C-4''), 159.0 (C-4), 175.7 (NH*C*OCH_2_). HRMS-ESI (*m/z*): calcd. for C_32_H_31_BrN_3_O_6_ [M−H]^−^, 632.1402; found 632.1411.

### 3.3. Typical Procedure for Bromination of Purine Nucleosides and Their Derivatives

5'-*O*-Monomethoxytrityl-*N*^2^-phenoxyacetylguanosine (**33**, 0.138 g, 0.2 mmol) was dissolved in aqueous DMF solution (H_2_O:DMF 1:4, 5 mL) under stirring. SMBI (1.1 equiv., 0.051 g, 0.22 mmol) was added at r.t. and the mixture stirred. Progress of the reaction was followed by TLC. An additional amount of the reagent (0.15 equiv., 0.007 g) was added into the reaction mixture after 1.5 h. On completion of the reaction by 2 h, the reaction mixture was filtered, evaporated to dryness under reduced pressure and coevaporated with water (2 × 2 mL). The crude reaction mixture was purified by column chromatography (4%–5% MeOH in DCM, v/v) to afford nucleoside **34** (0.148 g, 96%) in pure form as a white solid.

*8-Bromo-5'-O-monomethoxytrityl-N^2^-phenoxyacetylguanosine* (**34**). M.p. 136–139 °C; *R_f_* = 0.60 (10% methanol in dichloromethane); ^1^H-NMR (CD_3_OD): δ = 3.18 (d, *J* = 2.4 Hz, 0.5H, C-5'H_a_), 3.33 (dd, *J* = 10.0, 6.4 Hz, 1H, C-5'H_b_), 3.59 (s, 3H, OCH_3_), 4.04–4.09 (m, 1H, C-4'H), 4.44 (t, *J* = 5.6 Hz, 1H, C-3'H), 4.58 (d, *J* = 15.2 Hz, 1H, COCH_2(a)_O), 4.64 (d, *J* = 15.2 Hz, 1H, COCH_2(b)_O), 5.14 (t, *J* = 4.8 Hz, 1H, C-2'H), 5.91 (d, *J* = 4.4 Hz, 1H, C-1'H), 6.58 (d, *J* = 8.8 Hz, 2H, C-3'''H and C-5'''H), 6.86–6.91 (m, 3H, C-2''H, C-4''H and C-6''H), 7.01–7.06 (m, 6H, C-3''''H, C-4''''H, C-5''''H, C-3'''''H, C-4'''''H and C-5'''''H), 7.10 (d, *J* = 8.8 Hz, 2H, C-2'''H and C-6'''H), 7.18 (t, *J* = 8.0 Hz, 2H, C-3''H and C-5''H), 7.23–7.25 (m, 4H, C-2''''H, C-6''''H, C-2'''''H and C-6'''''H). ^13^C-NMR (CD_3_OD): δ = 55.7 (OCH_3_), 65.6 (C-5'), 68.3 (CO*C*H_2_O), 72.0 (C-2'), 72.8 (C-3'), 85.7 (C-4'), 87.6 (O*C*(Ar)_3_), 92.9 (C-1'), 113.9 (C-3''' and C-5'''), 116.1 (C-2'' and C-6''), 122.9 (C-5), 123.3 (C-4''), 127.4 (C-8), 127.9, 128.0, 129.6, 129.7, 130.8, 131.6 (C-2'', C-3'', C-5'', C-6'', C-2''', C-6''', C-2'''', C-3'''', C-4'''', C-5'''', C-6'''', C-2''''', C-3''''', C-4''''', C-5''''' and C-6'''''), 136.6 (C-1'''), 145.8, 146.0 (C-1'''' and C-1'''''), 148.3 (C-2), 151.1 (C-4), 156.3 (C-6), 159.0 (C-4'''), 160.1 (C-1''), 172.5 (NH*C*OCH_2_). HRMS-ESI (*m/z*): calcd. for C_38_H_33_BrN_5_O_8_ [M−H]^−^, 766.1518; found 766.1553.

## 4. Conclusions

In summary, we report here the development of an efficient and facile methodology for the bromination of nucleosides. We have applied this process successfully for both purine and pyrimidine nucleosides. Our process presents an efficient methodology of bromination for ribonucleosides, 2'-deoxynucleosides, silyl protected and DMTr/MMTr protected nucleosides. We believe this methodology may be applied for the bromination of a wide range of nucleoside derivatives.

## Supplementary Materials

Supplementary materials can be accessed at: http://www.mdpi.com/1420-3049/18/10/12740/s1.
