# Supplementary material for: An Efficient and Facile Methodology for Bromination of Pyrimidine and Purine Nucleosides with Sodium Monobromoisocyanurate (SMBI)
Source: Molecules. 2013 Oct 15;18(10):12740–50. doi: 10.3390/molecules181012740 (PMC6269699; doi:10.3390/molecules181012740)

Supplementary Materials

Figure S1. ^{1}H-NMR spectrum of **6** (DMSO-d_6, 400 MHz).

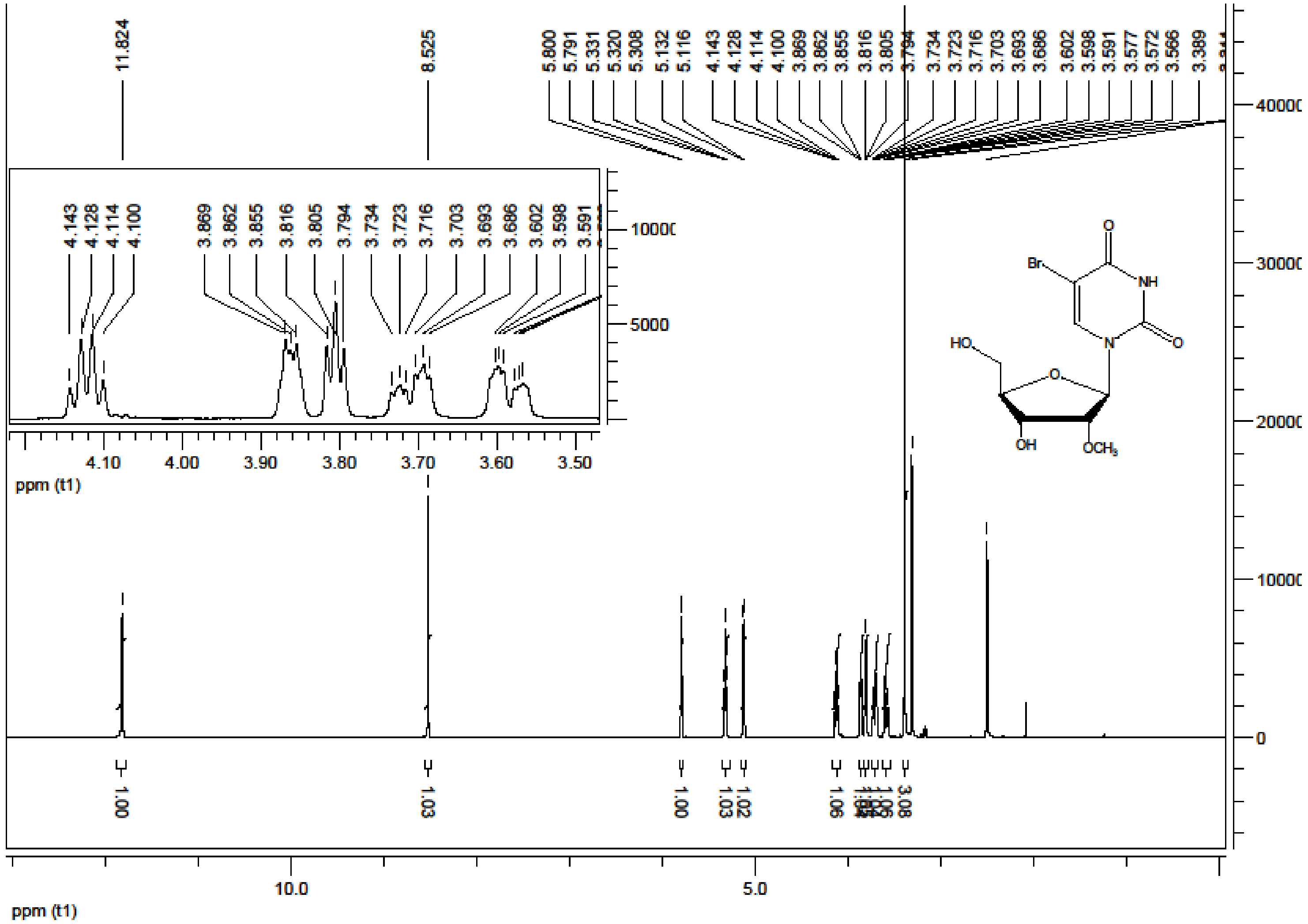

Figure S2. ^{1}H-^{1}H COSY spectrum of **6** in DMSO-d_6.

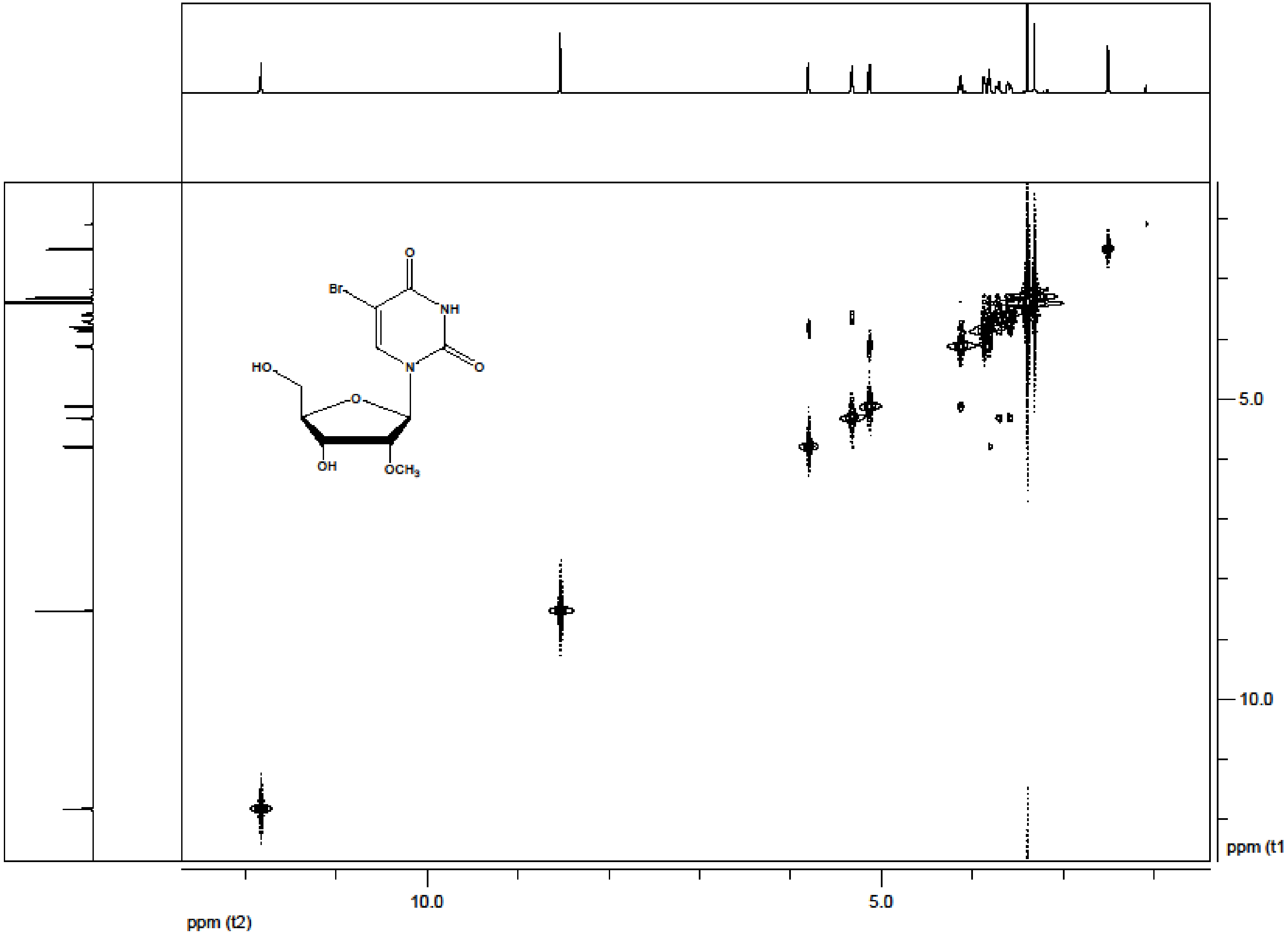

Figure S3. ^{13}C-NMR spectrum of **6** (DMSO-d_6, 100 MHz).

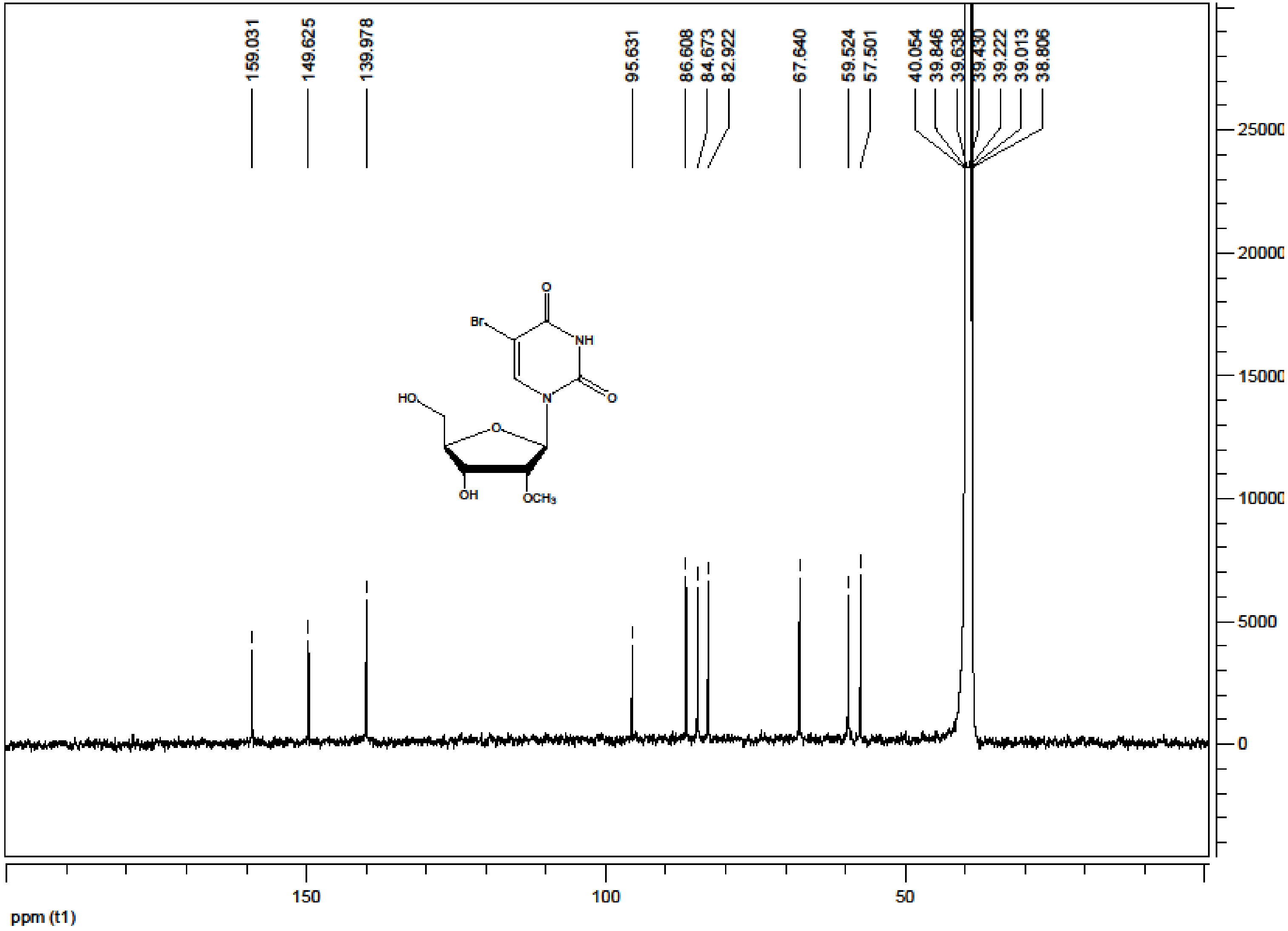

Figure S4. DEPT-135 spectrum of **6** (DMSO-d_6, 100 MHz).

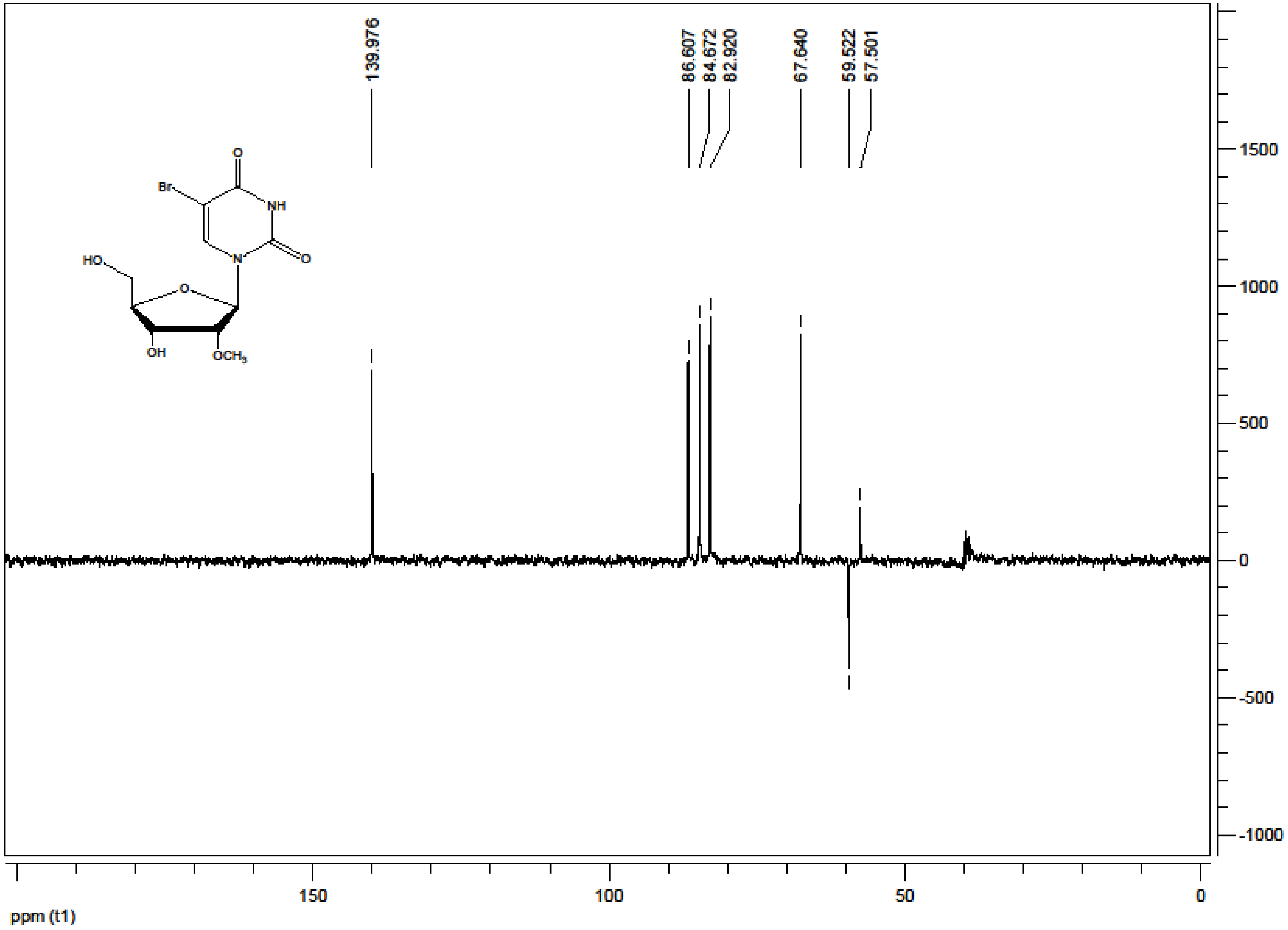

Figure S5. ^{1}H-NMR spectrum of **12** (CD_3OD, 400 MHz).

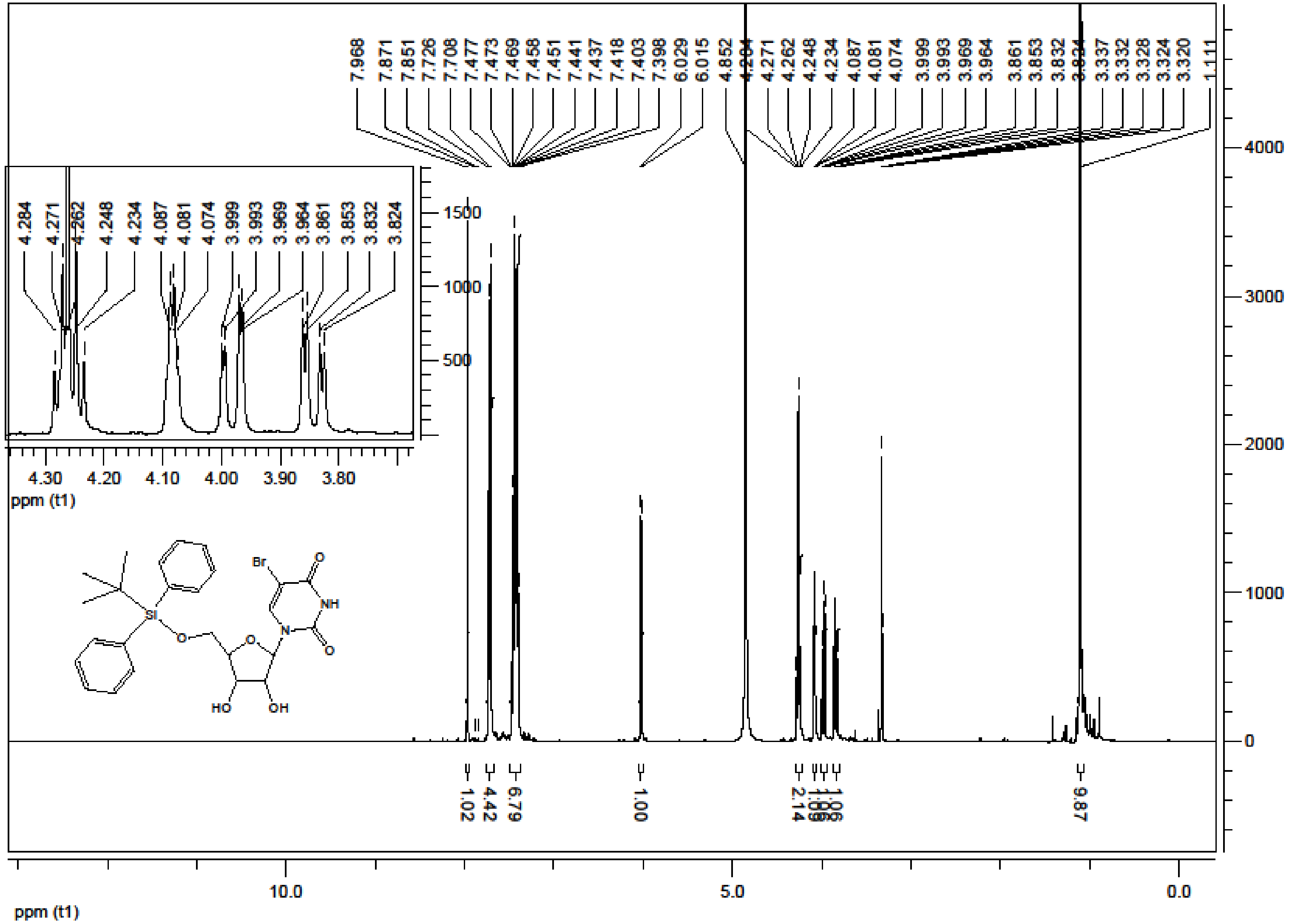

Figure S6. ^1H-^1H COSY spectrum of **12** in CD_3OD.

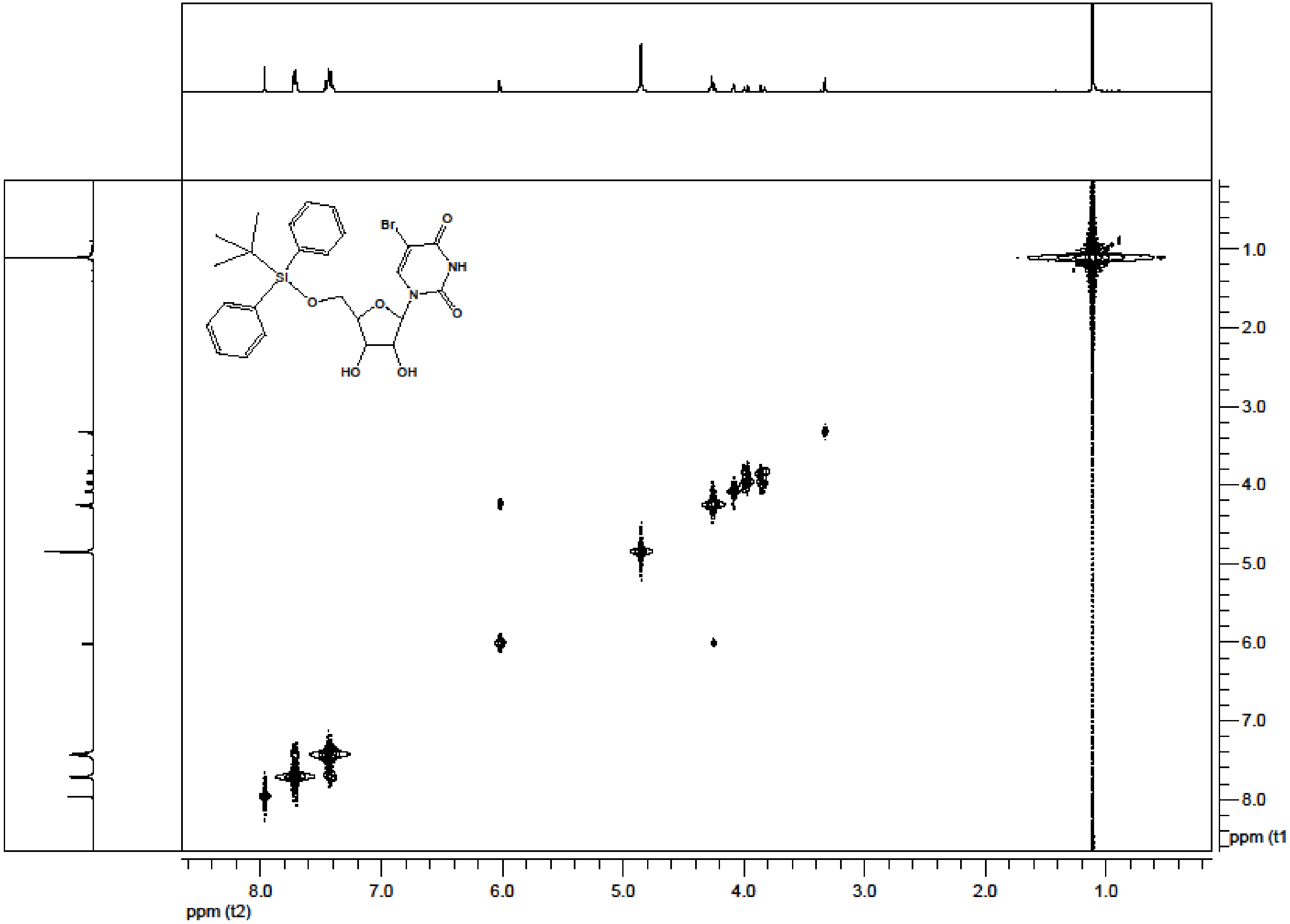

Figure S7. ^{13}C-NMR spectrum of **12** (CD_3OD, 100 MHz).

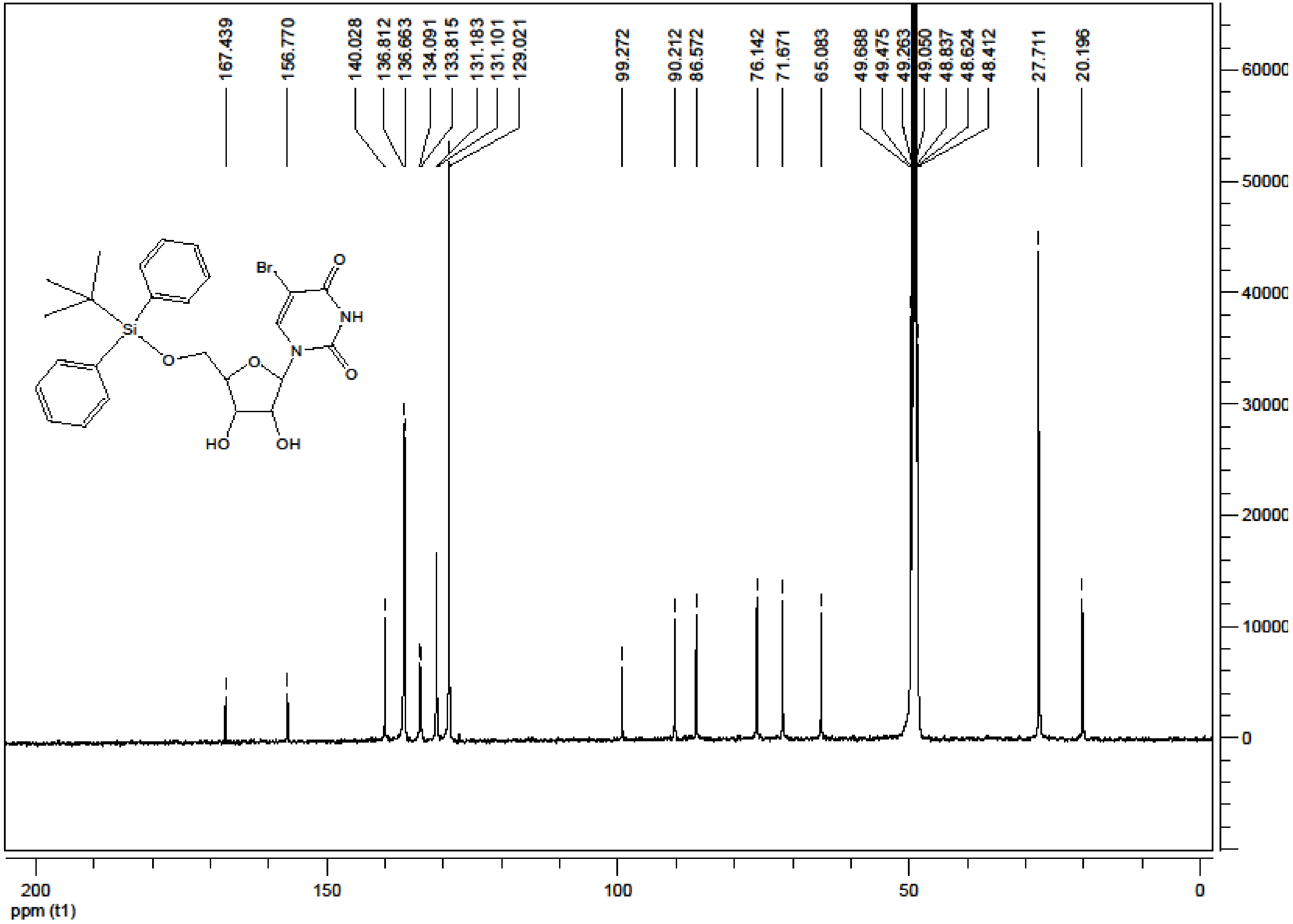

Figure S8. DEPT-135 spectrum of **12** (CD_3OD, 100 MHz).

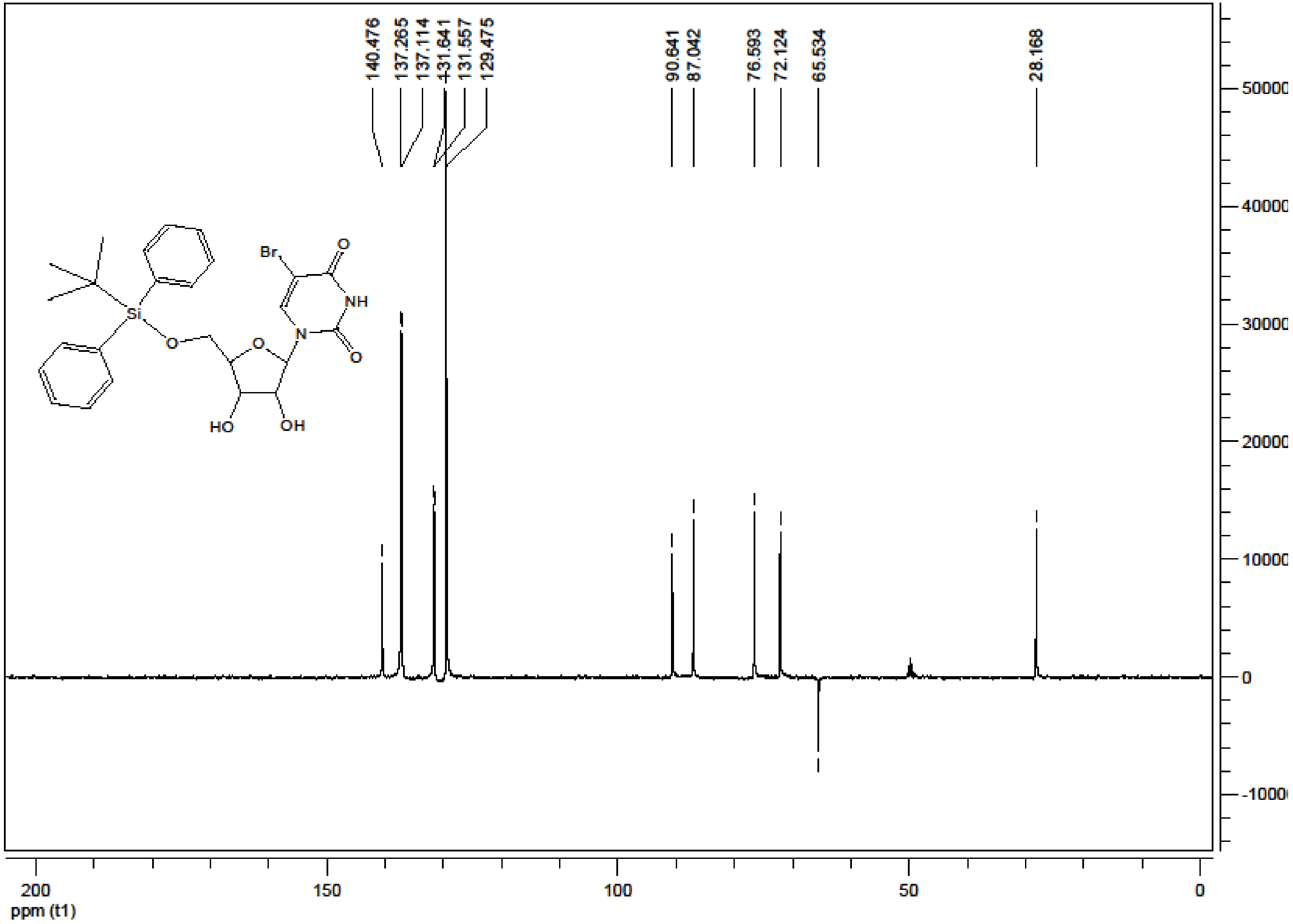

Figure S9. ^{1}H-NMR spectrum of **18** (D_2O, 400 MHz).

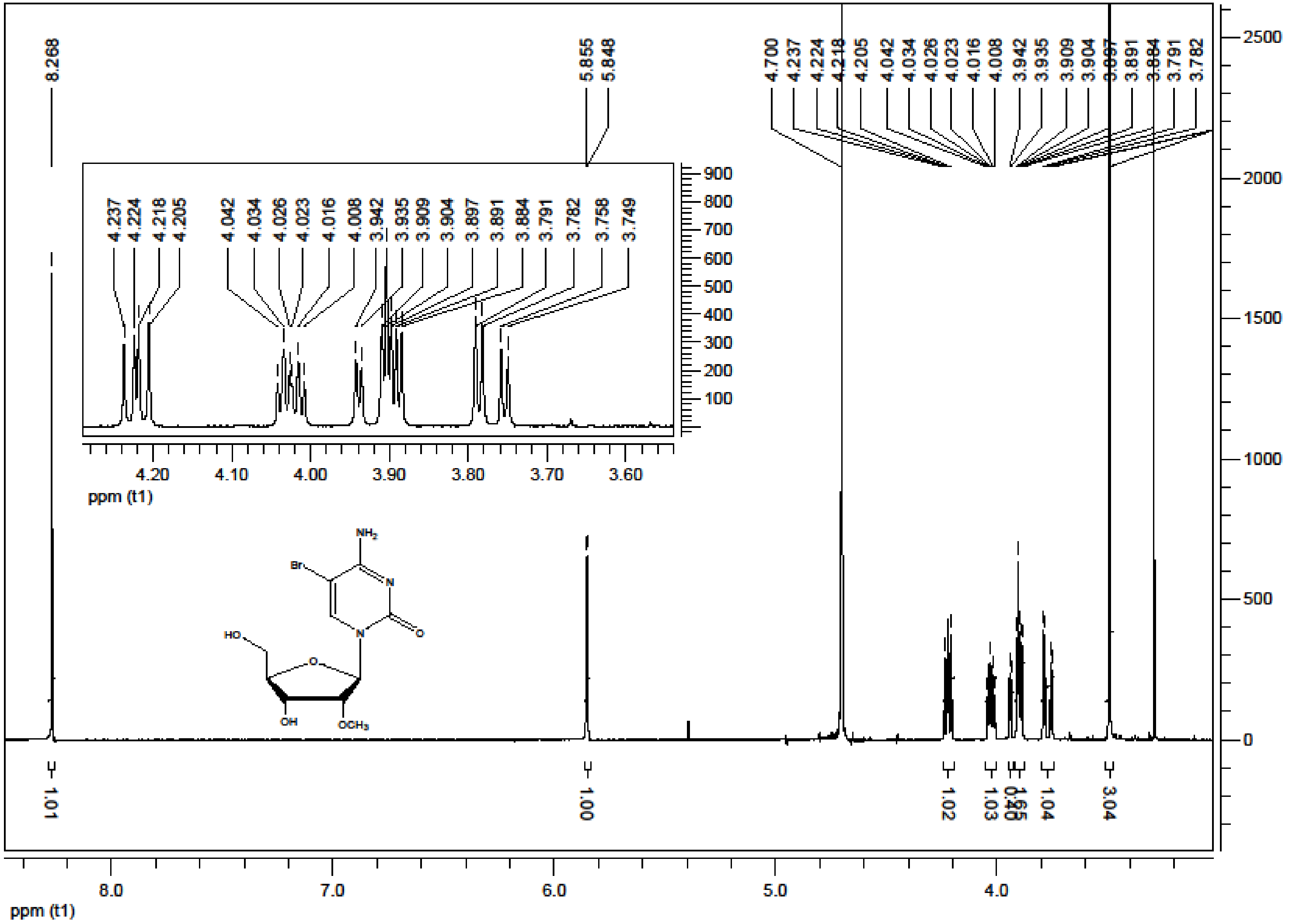

Figure S10. ^1H-^1H COSY spectrum of **18** in D_2O.

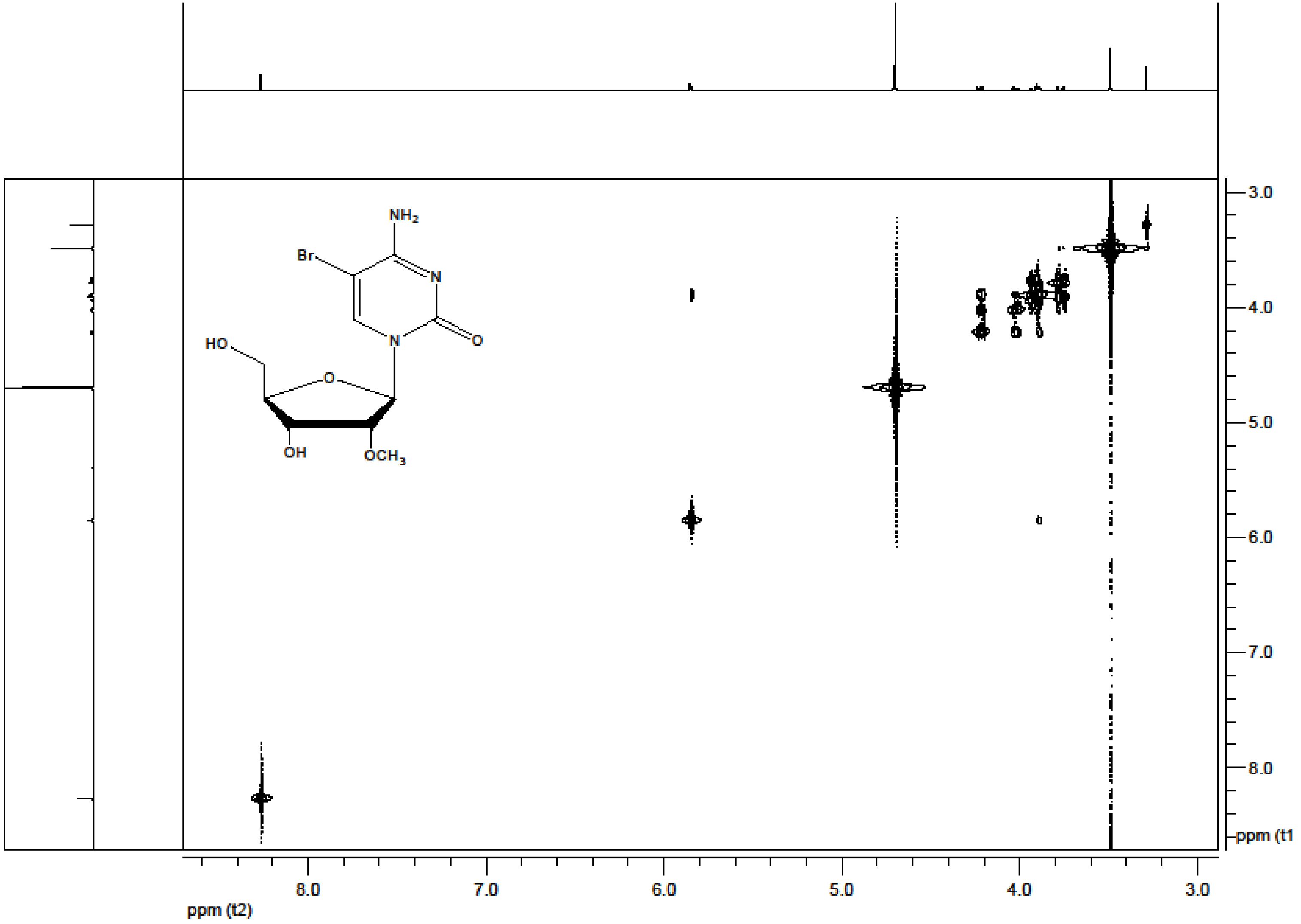

Figure S11. ^{13}C-NMR spectrum of **18** (D_2O, 100 MHz).

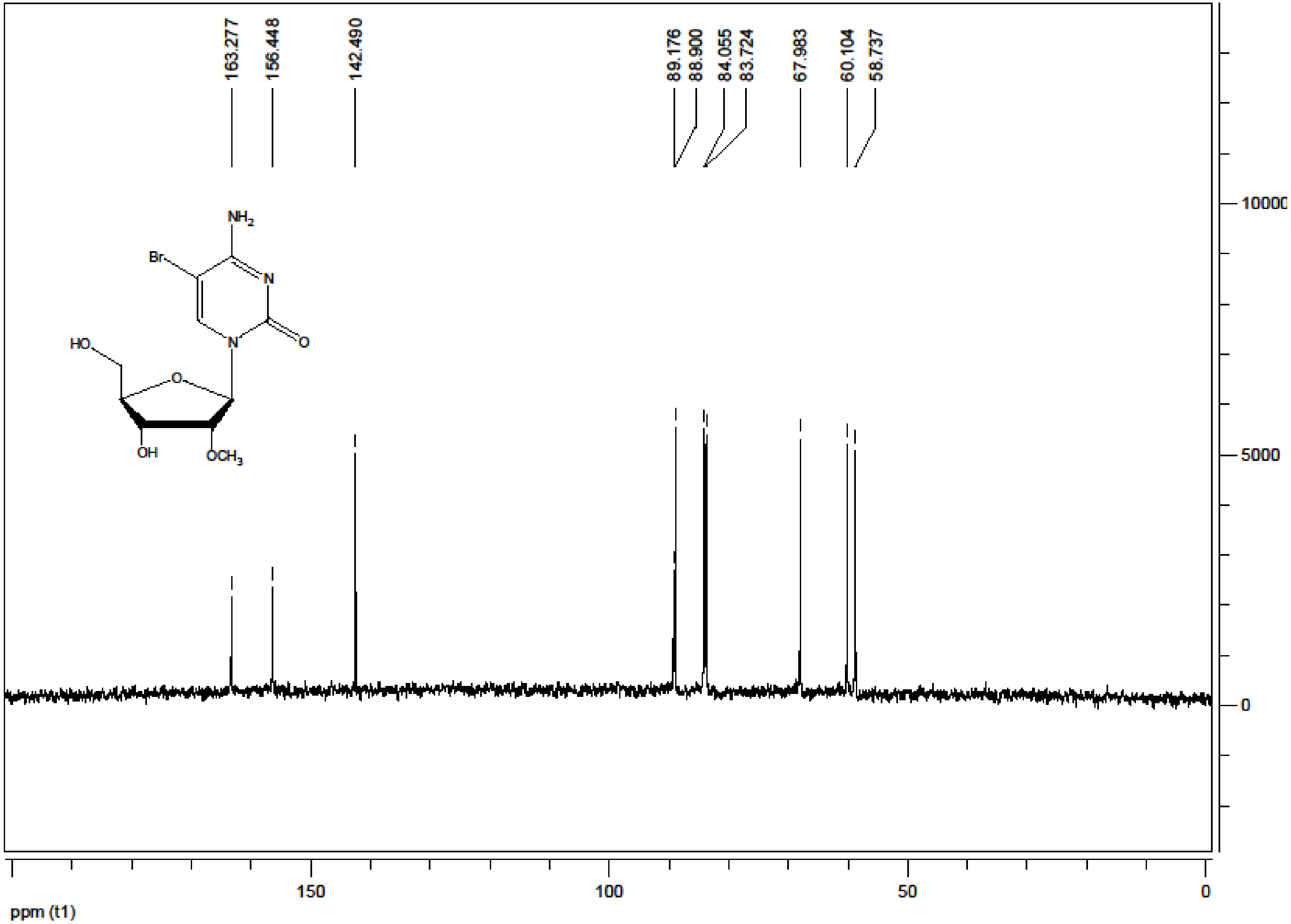

Figure S12. DEPT-135 spectrum of **18** (D_2O, 100 MHz).

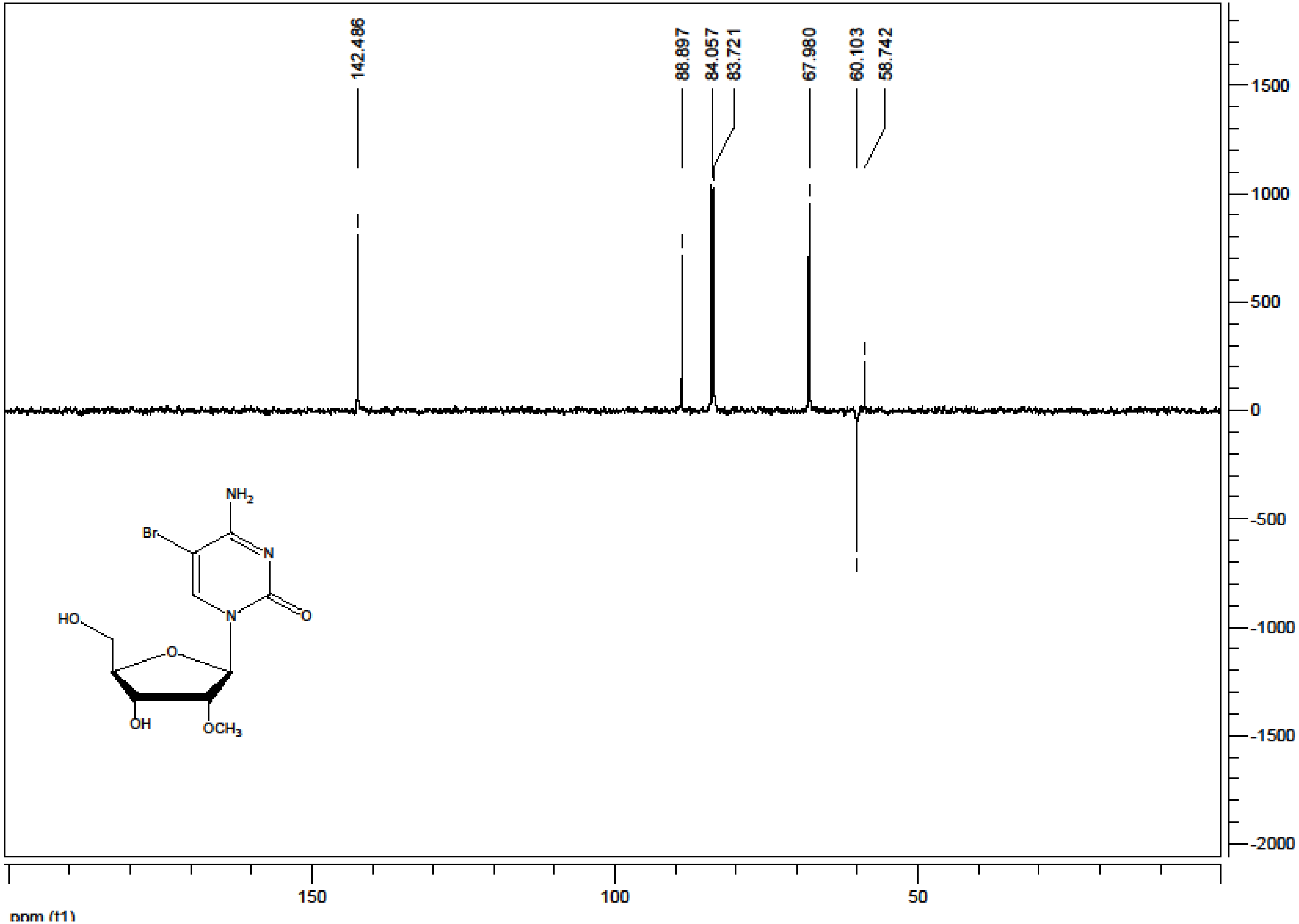

Figure S13. ^{1}H-NMR spectrum of **20** ($CDCl_3$, 400 MHz).

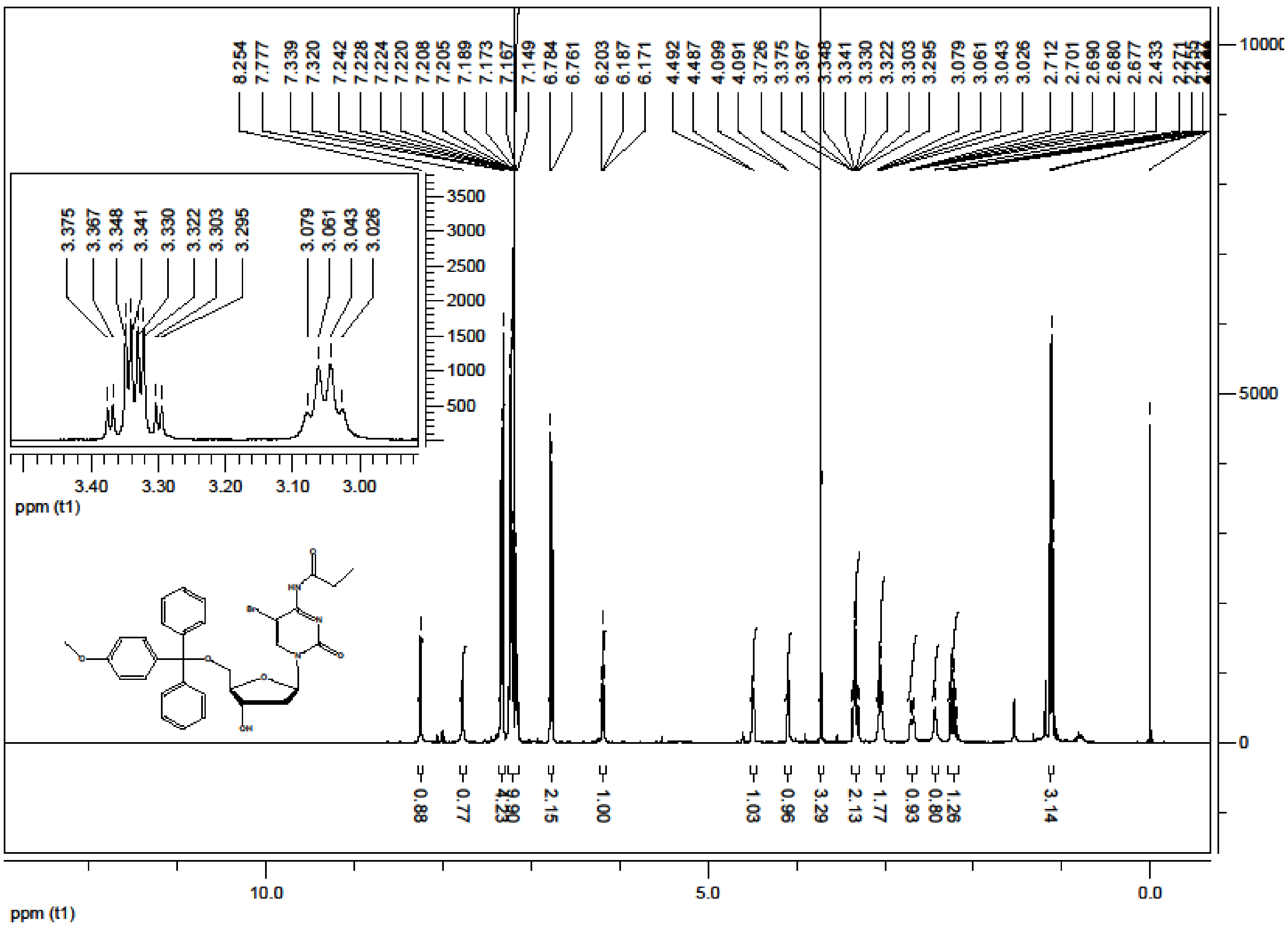

Figure S14. ^{1}H-^{1}H COSY spectrum of **20** in $CDCl_3$.

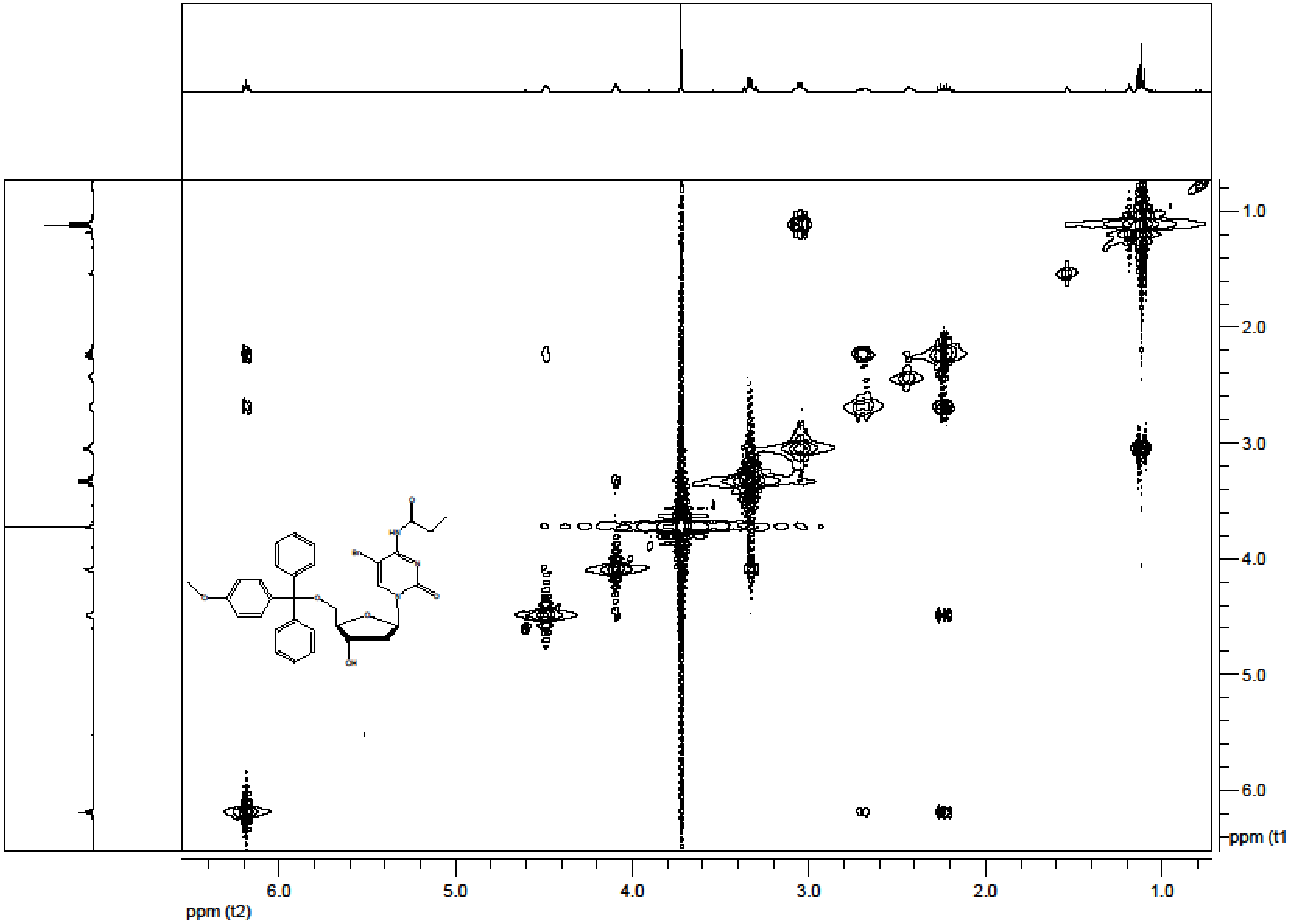

Figure S15. ^{13}C-NMR spectrum of **20** ($CDCl_3$, 100 MHz).

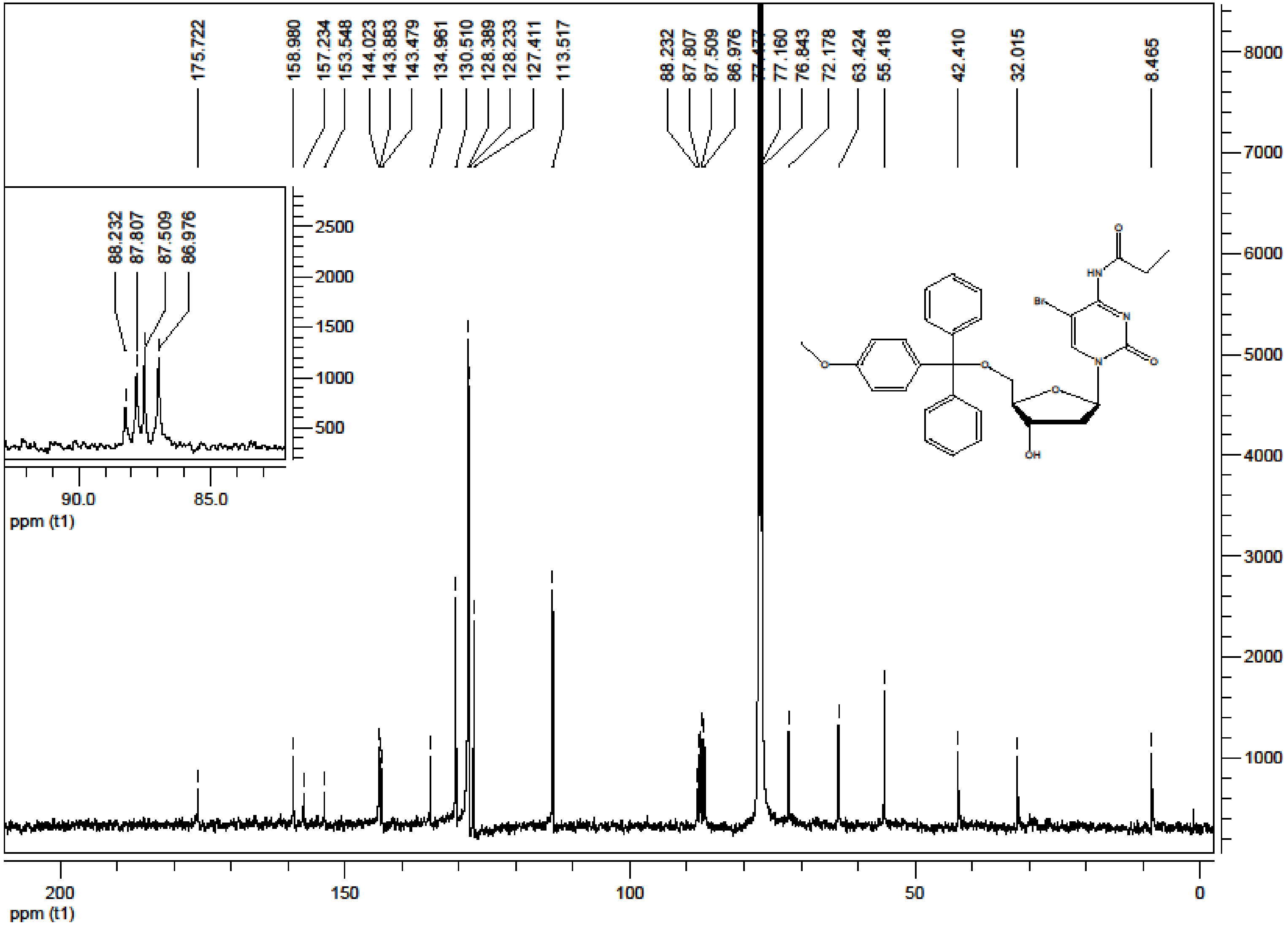

Figure S16. DEPT-135 spectrum of **20** ($CDCl_3$, 100 MHz).

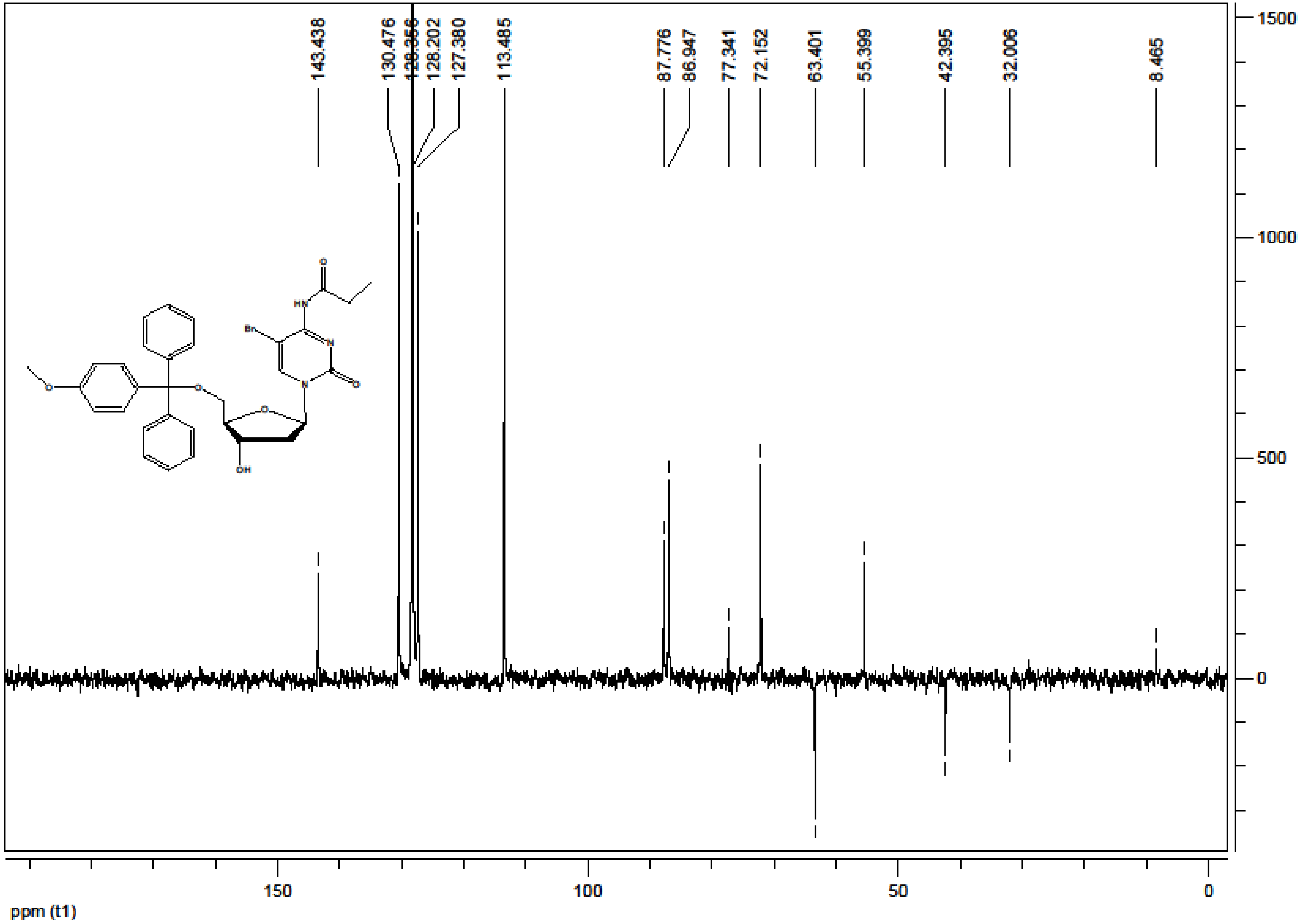

Figure S17. ^{1}H-NMR spectrum of **34** ($CDCl_3$, 400 MHz).

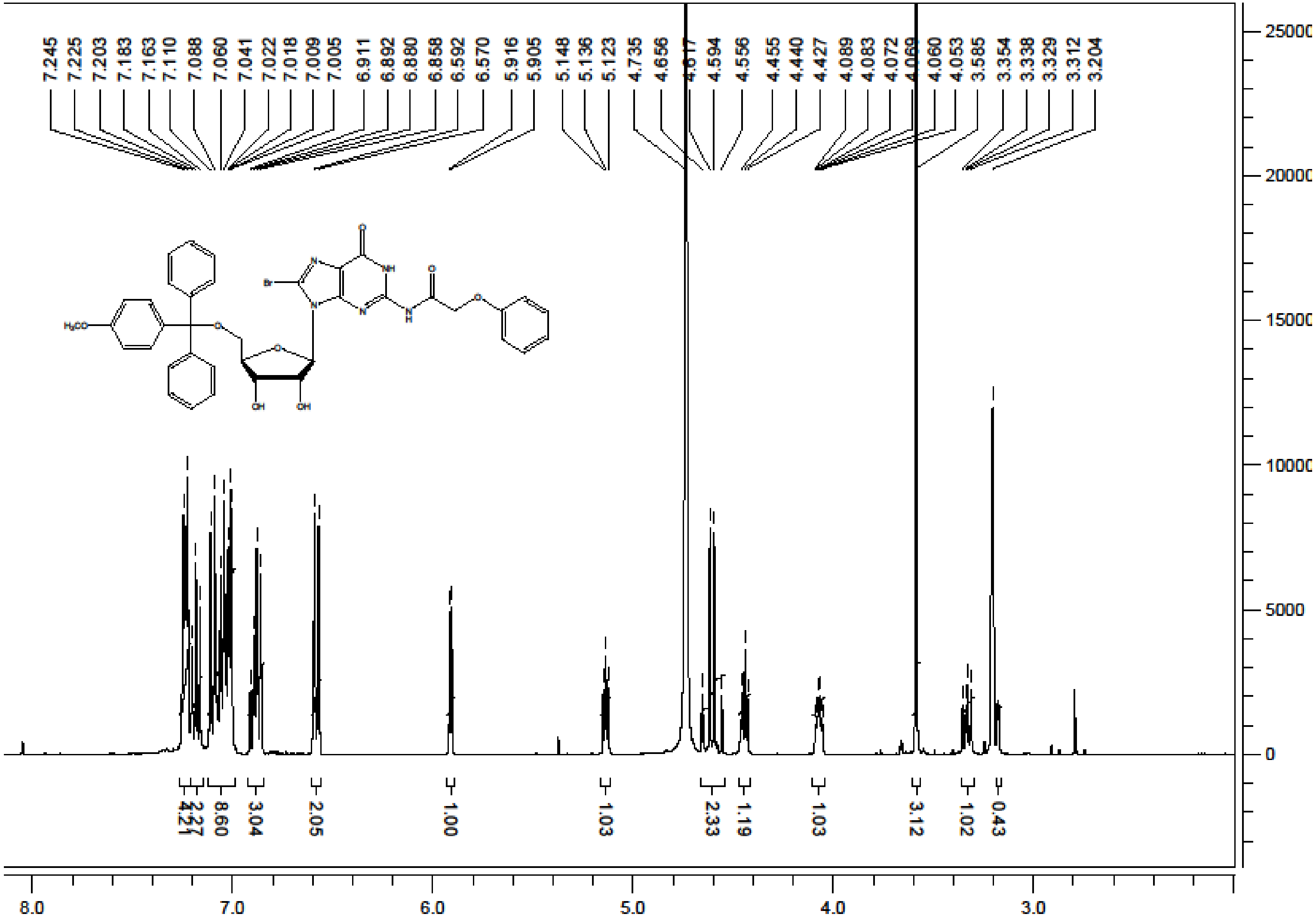

Figure S18. ^{1}H-^{1}H COSY spectrum of **34** in $CDCl_3$.

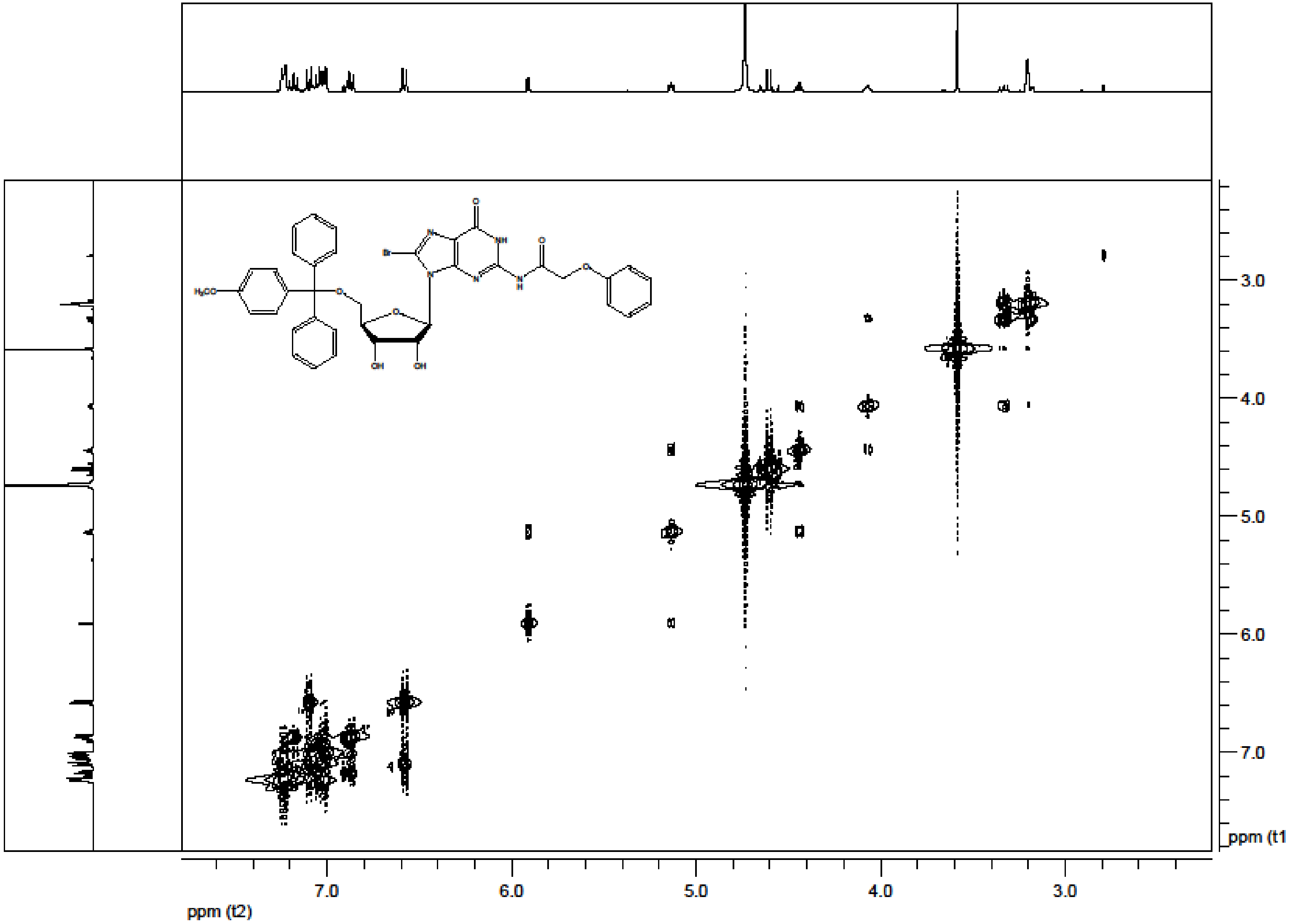

Figure S19. ^{13}C-NMR spectrum of **34** ($CDCl_3$, 100 MHz).

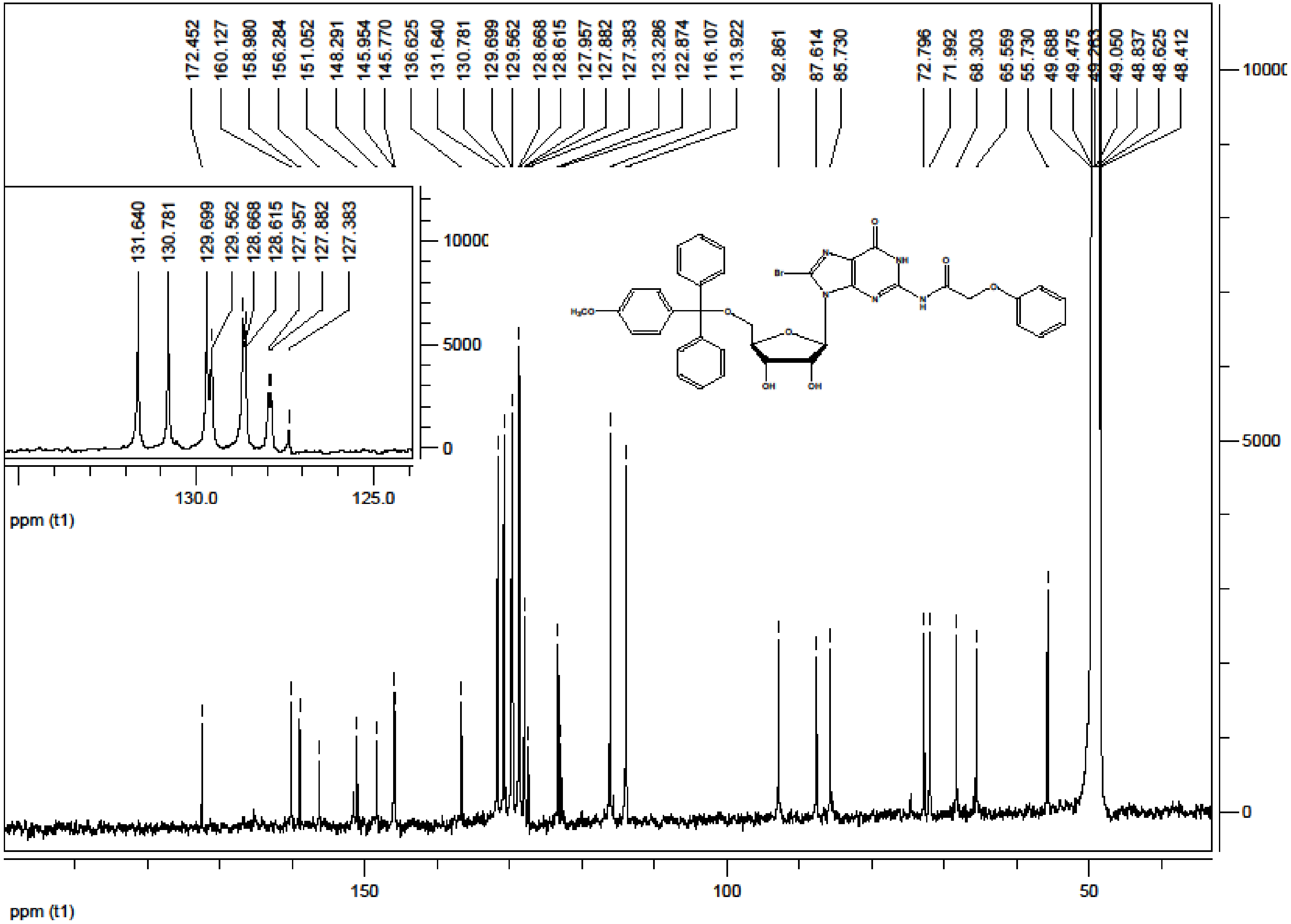

Figure S20. DEPT-135 spectrum of **34** ($CDCl_3$, 100 MHz).

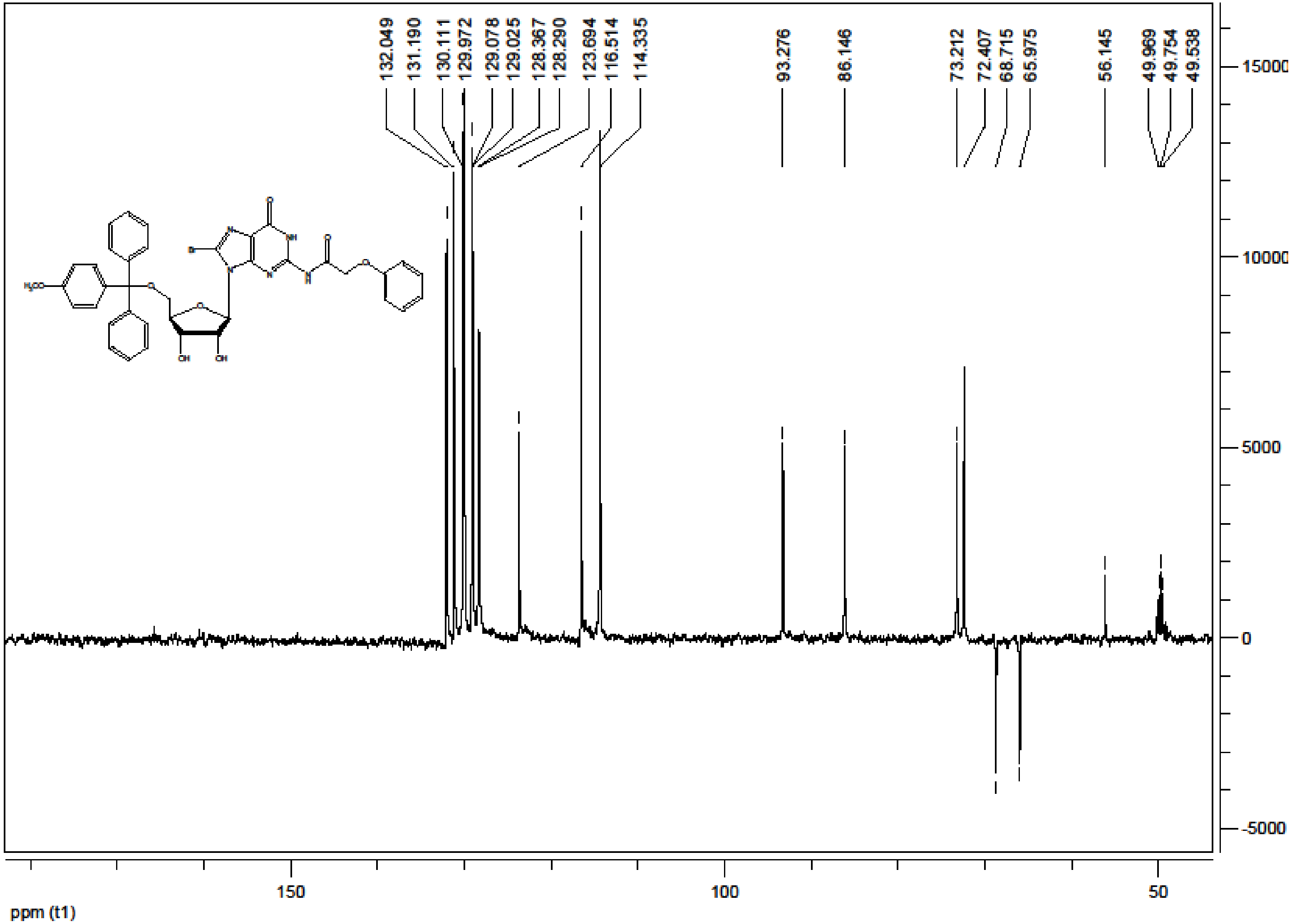

Supplement: Supplementary file 1 [file molecules-18-12740-s001.pdf]
